# Assessing clinically relevant endemic environmental bacterial and viral contamination in neonatal intensive care areas

**DOI:** 10.1017/ash.2026.10332

**Published:** 2026-05-11

**Authors:** Bobby G. Warren, Amanda M. Graves, Aaron Barrett, Guerbine Fils-Aime, Isadora Mamikunian, Melissa Campbell, Lakshmi Katakam, Deverick J. Anderson, Ibukunoluwa C. Kalu

**Affiliations:** 1 Disinfection, Resistance, and Transmission Epidemiology (DiRTE Lab), Durham, NC, USA; 2 Duke Center for Antimicrobial Stewardship and Infection Prevention, Durham, NC, USA; 3 Division of Pediatrics Infectious Diseases, Duke University Medical Center, Durham, NC, USA

## Introduction

Healthcare-associated infections (HAIs) in the neonatal intensive care unit (NICU) are among the most serious threats to infant survival. NICU patients are highly susceptible to HAIs due to immature immunity, invasive procedures, and prolonged hospitalizations.^1^ Reported NICU HAI rates range from 8% to 15%, markedly higher than the ∼3% prevalence among adult hospital admissions, and up to 40% among extremely low birthweight infants, corresponding to an estimated 28,000–52,500 NICU-associated infections annually in the United States.^2–5^

Despite this burden, few studies have prospectively evaluated environmental contamination in NICUs. Most existing data are from outbreak settings, leaving fundamental gaps in knowledge. No prior work has examined how pod size and layout, as well as shared and patient-dedicated surfaces, influence contamination risk. As a result, infection prevention practices are largely adapted from adult care models. We conducted a pilot study to characterize bacterial and viral contamination of high-touch surfaces in NICU environments by surface type and pod size.

## Methods

We conducted a prospective, pilot study in a 74-bed, level 4 NICU at a tertiary academic medical center. During a two-week period in April 2025, high-touch surfaces were sampled from multiple pods in the NICU representing both shared and patient-dedicated spaces. Enrolled pods housed 2, 4, 8, or 15 patient beds. Pods and surfaces received routine cleaning per unit policy.

Surfaces were grouped into three categories: patient-specific surfaces (PSSs), shared patient surfaces (SPSs), and unit-wide surfaces (UWSs). PSSs included isolette doors and handles, isolette drawer handles, isolette rails, bottle warmers, IV poles, temperature probes, keyboards, and family chairs. SPSs included breast pumps, clinician phones, diaper scales, glucometers, room tables, and handwashing sinks. UWSs included blanket warmers and breastmilk refrigerator handles.

Surfaces were sampled with flocked swabs premoistened in neutralizing buffer. Samples were analyzed for total bacterial bioburden and clinically important pathogens (CIPs), with bacterial identification by culture on selective media and viral detection by qPCR.^6,7^ Bacterial CIP included *Staphylococcus aureus*, *Enterococcus spp.*, gram-negative rods, and *Clostridioides difficile*. Viral CIP targets included adenovirus and respiratory syncytial virus (RSV). Descriptive statistics summarized bioburden and detection frequency by organism, surface, and pod.

## Results

Ninety-nine surface samples were collected during the study. Of these, 74 (75%) were from PSSs, 19 (19%) SPSs, and 6 (6%) UWSs. Samples represented pods of varying sizes: 11 (11%) from two-bed pods, 15 (15%) from four-bed pods, 30 (30%) from eight-bed pods, and 37 (38%) from fifteen-bed pods.

**Figure 1.**
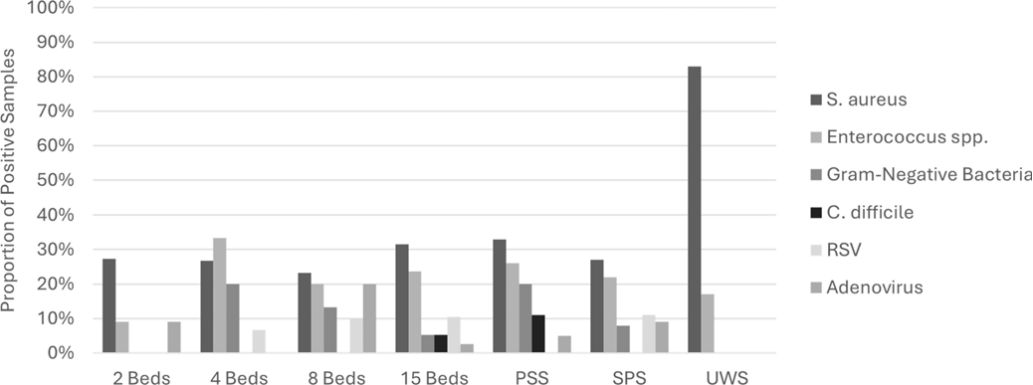
Proportion of sampled surfaces positive for ≥1 study pathogen, stratified by pod size and surface category. PSS, patient-specific surface; SPS, shared patient surface; UWS, unit-wide surface.

The median bioburden across all surfaces was 130 CFU (IQR, 30–660), with no significant differences between pod sizes or surface categories (data not shown). Overall, 56 (57%) samples were positive for at least one CIP. Several surfaces were contaminated in all sampled instances with at least one CIP including isolette rails, family chairs, isolette drawer handles, handwashing sinks, diaper scales, room tables, breast pumps, and breastmilk refrigerator handles. By surface category, 41 (55%) of PSS, 10 (53%) of SPS, and 5 (83%) of UWS samples were positive for any study pathogen and 20 (27%) of PSS, 6 (33%) of SPS, and 5 (83%) of UWS samples for bacterial contamination (Figure 1). Viral contamination was uncommon and largely confined to PSSs, which accounted for 15 of 16 (94%) total viral detections.

When stratified by pod size, contamination was identified in 4 (36%) samples from two-bed pods, 9 (60%) from four-bed pods, 15 (50%) from eight-bed pods, and 21 (55%) from fifteen-bed pods, with no statistically significant differences (Figure 1). Overall, *S. aureus* (31%) and *Enterococcus spp.* (22%) were the most frequently recovered bacteria, followed by gram-negative rods (9%) and *Clostridioides difficile* (2%). None of the 34 infants receiving routine care in sampled rooms had documented infections or positive clinical cultures in the preceding 30 days.

## Discussion

Despite low overall bioburden and no documented patient infections, more than half of sampled NICU surfaces harbored at least one CIP. *S. aureus and Enterococcus* spp. predominated, consistent with their persistence in healthcare environments.^8,9^ High-touch items such as isolette components, sinks, and shared clinical equipment were contaminated in all sampled instances, highlighting potential persistent reservoirs for pathogen transmission in neonatal settings. Viral detections were almost exclusively limited to PSSs, suggesting localized contamination associated with direct caregiving activities, which aligns with the shorter environmental persistence of most respiratory and enteric viruses relative to bacteria and the possibility of prolonged or asymptomatic viral shedding in hospitalized infants. These findings underscore the role of spatial layout and pod configuration in contamination patterns seen in NICUs. However, given the small scale of this pilot study, differences by pod size or surface type may not have been fully captured. This study provides an early framework for linking unit design with infection risk, an area not previously explored in neonatal infection prevention research.

Limitations include the single-center design, limited sampling, and potential under- or overestimation of contamination due to culture-based and qPCR methods.^10^ Despite these limitations, the study demonstrates the feasibility of systematic environmental surveillance in neonatal settings.

These findings highlight that environmental contamination with CIPs is common in NICUs, even in the absence of known patient infections. This pilot represents an initial step toward understanding contamination dynamics unique to neonatal care. Larger, multicenter studies are needed to identify drivers of contamination across NICU layouts and workflows. Current infection-prevention protocols, largely adapted from adult settings, may not fully address neonatal care environments. Future work should focus on developing neonatal-specific prevention strategies and cleaning practices that reflect the realities of modern NICU care.
